# Changing the research paradigm for digital transformation in healthcare delivery

**DOI:** 10.3389/fdgth.2022.911634

**Published:** 2022-09-06

**Authors:** Elizabeth A. Regan

**Affiliations:** Department of Integrated Information Technology, College of Engineering and Computing, University of South Carolina, Columbia, SC, United States

**Keywords:** digital transformation, healthcare delivery science, healthcare fragmentation, design science, convergence research, transdisciplinary research, translational research

## Abstract

The growing focus on healthcare transformation (i.e., new healthcare delivery models) raises interesting issues related to research design, methodology, and funding. More than 20 years have passed since the Institute of Medicine first called for the transition to digital health with a focus on system-wide change. Yet progress in healthcare delivery system change has been painfully slow. A knowledge gap exists; research has been inadequate and critical information is lacking. Despite calls by the National Academies of Science, Engineering, and Medicine for convergent, team-based transdisciplinary research with societal impact, the preponderance of healthcare research and funding continues to support more traditional siloed discipline research approaches. The lack of impact on healthcare delivery suggests that it is time to step back and consider differences between traditional science research methods and the realities of research in the domain of transformational change. The proposed new concepts in research design, methodologies, and funding are a needed step to advance the science. The Introduction looks at the growing gap in expectations for transdisciplinary convergent research and prevalent practices in research design, methodologies, and funding. The second section summarizes current expectations and drivers related to digital health transformation and the complex system problem of healthcare fragmentation. The third section then discusses strengths and weaknesses of current research and practice with the goal of identifying gaps. The fourth section introduces the emerging science of healthcare delivery and associated research methodologies with a focus on closing the gaps between research and translation at the frontlines. The final section concludes by proposing new transformational science research methodologies and offers evidence that suggests how and why they better align with the aims of digital transformation in healthcare delivery and could significantly accelerate progress in achieving them. It includes a discussion of challenges related to grant funding for non-traditional research design and methods. The findings have implications broadly beyond healthcare to any research that seeks to achieve high societal impact.

## Introduction

Over two decades have passed since the Institute of Medicine recognized the growing problems in the U.S. healthcare delivery system and called for the transition to digital health with a focus on system-wide change (1). However, progress in healthcare delivery system change has been painfully slow (2–10). Despite calls by the National Research Council (11) and the National Academies of Science and Medicine (12) for convergent, team-based transdisciplinary design science research, the preponderance of healthcare research and funding continues to support more traditional disciplinary research approaches (5, 13, 14). The emerging science of healthcare delivery holds promise, but has it advanced enough? Does it appropriately consider the complexity of systemic change? Is it time to step back and consider differences between traditional science research methods and the realities of research in the domain of transformational change? The proposed new concepts in research design, methodologies, and funding are a needed step to advance the science.

The purpose of this research is to examine the growing gap between expectations and demands for creating a 21^st^ century healthcare delivery system and the current methodologies, practices, and funding available to address them—what works, what does not, and why. The approach takes a deep transdisciplinary view. The underlying conceptual frameworks span healthcare delivery science, design science/design thinking, human-centered design/user experience, complex systems science, social sciences– especially organizational development, change leadership, interorganizational systems, business process management, healthcare service management, value cocreation, digital transformation, IT adoption and assimilation, implementation science, medical practice, comparative effectiveness research, translational research, and learning organization theory–only a selection of the most relevant of which are discussed in ensuing sections and referenced in the bibliography. The next section summarizes current expectations and drivers related to digital health transformation. The third section then discusses strengths and weaknesses of current research and practice with the goal of identifying gaps. The fourth section on research methodologies then synthesizes this information with the aim of suggesting critical success factors for closing the gaps between research and translation at the frontlines. The final section on changing the research paradigm concludes by proposing new transformational science research methodologies and offers evidence that suggests how and why they better align with the aims of digital transformation in healthcare delivery and could significantly accelerate progress in achieving them. The findings have implications broadly beyond healthcare to any research that seeks to achieve high societal impact.

## The problem of healthcare fragmentation

Healthcare delivery transformation is a truly complex socio-technical systems problem at the intersection of multiple disciplinary traditions (10, 15–17). Significant issues in U.S. healthcare delivery persist despite advances in technologies, medicine, health policies, regulations, and resources (2, 5, 18, 19). Many Big Tech healthcare initiatives have ended in failure, stifling true innovation in healthcare delivery and reducing market confidence in technologies that are successful in addressing real market needs (10). We essentially have the greatest medical science in the world with a 19^th^ Century delivery system incapable of providing consistent, affordable care for all citizens (20). The term healthcare “delivery system” is used broadly to include the wide range of care givers and venues that provide clinical care services, all of which operate largely in silos with disjointed coordination among them leading to errors, gaps, duplication of services, and all too often, poor patient outcomes. In their documentation of the problem, the Institute of Medicine (IOM) attributes >100,000 deaths a year to preventable medical errors, resulting from system lapses that fail to deliver well-established standards of practice. The IOM findings and recommendations were issued in 2000, and the statistics have not improved (4, 5, 21–28). The U.S. healthcare system faces a daunting set of challenges, including high costs, disparities in access, and significant gaps between scientific evidence and actual practice (5, 21, 28–30). Healthcare costs are projected to continue rising at a rate faster than growth in the overall economy, with expenditures increasing from $3.6 trillion in 2018 to nearly $6.0 trillion by 2027 (31). According to estimates, up to one-third of healthcare spending in the US each year may be classified as waste that relates largely to failures of care delivery, care coordination, and overuse (10, 32, 33). Chronic illness accounts for 75% of total health system costs in the U.S. (34, 35). Yet the delivery system remains focused primarily on acute care and not chronic care (5, 36, 37). Research by RAND Corporation indicates that patient care meets recommended evidenced-based standards of care only 55 percent of the time (13). Despite these concerns, <0.1% of total healthcare spending in the U.S. goes toward research to improve how we deliver care, which represents only 3.6% of the NIH budget (24, 38, 39).

The research on quality, safety, and coordination issues indicates that most barriers to transforming healthcare delivery are neither medical problems nor individual performance problems, but rather they are *complex system problems* (1, 5, 40–42). The problems have grown increasingly worse and more expensive with an aging population and high incidence of multiple chronic conditions. The explosion of technology innovations and nontraditional market entrants may be further exacerbating the fragmentation problem.

The siloed nature of healthcare in an uncoordinated fashion across multiple specialties and settings, presents one of the greatest challenges limiting delivery system optimization (13, 14). Gaps impeding solutions include problems in both clinical and technical domains and especially at the intersection of the two. Our fragmented U.S. healthcare delivery system is a major driver of spiraling cost increases, inequities, and deteriorating patient outcomes (5, 14, 41, 43–47). It is no fluke that the technologically advanced but fragmented U.S. healthcare system ranks highest in cost but 37^th^ globally in performance (14, 48). Spending more on parts has not improved the whole (14). An abundance of digital innovations and research has generated much excitement, but relatively minor impact on how healthcare is delivered (39, 49). The inability to address this fragmentation problem has been attributed largely to our narrowly focused approaches with insufficient attention to the whole (5, 14, 49–51). U.S. healthcare is built on an acute care paradigm that has grown increasingly fragmented and inefficient at addressing the chronic care and other health needs of today's population (14).

The problem U.S. healthcare faces is how to leverage advanced technologies and information sharing to transform the healthcare ecosystem from an underperforming conglomeration of independent entities (individual practitioners, small group practices, clinics, hospitals, pharmacies, community health centers, public health agencies, etc.) into a high-performance system in which participants recognize their interdependence and the implications and repercussions of their actions on the system as-a-whole (14, 51, 52). Moreover, lessons learned from multiple failures among Big Tech innovation ventures have demonstrated that the healthcare industry is a different animal with intricate, deep-rooted problems that cannot be solved with a one-size-fits-all approach; but rather that improving healthcare requires specialized knowledge and applications to transform processes from the inside out (10). These problem have been successfully addressed by many other industries greatly expanding their capabilities to meet customer needs (e.g., banking, airlines, retail, and others). Although healthcare communities across the nation have made some progress in implementing and using health IT to share relevant patient information, the lack of widespread adoption of reliable systems that can share and integrate communication across institutional and organizational boundaries continues to hinder efforts to improve care delivery (5, 8).

Nationally, healthcare and public health institutions are ill prepared to meet healthcare changes required by the *21*^*st*^ *Century Cures Act* and national priorities for a fully connected healthcare system that empowers patients, caregivers, and their healthcare providers to access, exchange, and use digital health information (8). Sharing patient information remains problematic, even within health systems, and sharing information across institutions is extremely limited. The COVID-19 pandemic of 2020 shed light on shortcomings in our ability to share information to coordinate public health. From a patient perspective, hand-offs between silos present many challenges for coordination of care leading to delays and errors (2, 5, 53, 54). The lack of widespread adoption of a reliable system that can share and integrate communication across institutional boundaries is a significant barrier to improving care coordination (55). Our abilities to use and learn from the vast amounts of patient data now available in digital format have barely scratched the surface (8). Work to implement data standardization (US Core Data for Interoperability) and a national health information exchange network (QHINS and TEFCA) called for in the 21^st^ C. *Cures Act of 2017 are slowly moving forward*. However, little funding or support has been allocated for research or integration at the frontlines of care delivery.

Currently, consensus around what an optimized U.S. healthcare delivery system would look like is lacking, but there is general agreement around the following objectives for optimization: [(8, 56), Fed Health IT Strategic Plan].

- Interoperability for seamless sharing of data across systems and among health institutions of all types.
- Patient-centered–organized around patient needs rather than optimized around provider organizations (putting individuals first).
- Focus on quality of care, safety, reduction of medical errors, and improved efficiency.
- Transition to Pay-for-Value (from Fee-for-Service)–a better aligned payment system.
- Coordination across the entire continuum of patient care.
- Connect healthcare with health data (Need for patient electronic health data always available at the point-of-care).
- Need to shift from episodic care to population health focus.
- Team-based care.
- Equitable access.
- Put research into action: Strengthen feedback loops between scientific, public health, and healthcare communities to efficiently translate evidence into clinical practice and improvement (“Learning Healthcare Organizations”) (56–58).

Essentially, each of these objectives for transformation represents a significant paradigm shift from the status quo. The shift in mind set and organizational culture required to implement solutions is significant and often under appreciated. Problems in healthcare delivery are well documented, but why is progress in addressing them so slow and difficult? And, most importantly, what can we do about it? These are critical research questions, which remain largely unaddressed.

## Research design for healthcare delivery transformation

A growing body of evidence reveals that what we are doing now has not been working. In many respects, paper silos have now been replaced with digital silos, but progress toward substantive changes in healthcare delivery have been minimal. Much of the research remains in the realm of innovation outside of the core processes that drive care delivery (49, 59–61). The research literature related to transforming healthcare delivery reveals many challenges, not the least of which is the complexity of the U.S. healthcare ecosystem. The dynamics of the healthcare ecosystem are highly regulated, especially in the clinical environment. Despite recent trends for hospitals to employ physicians, many physicians and physician groups remain autonomous. Thus, hospitals generally experience strong cultural tensions between the clinical and administrative environments. Although operational units function essentially as silos, they are at the same time highly interdependent. Since healthcare is largely subject to a third-party payer system, it does not operate on a true market economy. As thought leader Dr. Don Berwick, former administrator of the Centers for Medicare and Medicaid, President Emeritus of Institute for Healthcare Improvement and Harvard Professor, described it, the U.S.is saddled with “... a viciously complicated legacy payment system designed by no one at all” (3). There is little price transparency, and in most cases, consumers of healthcare do not know the cost of what they are buying—at least not until well after the fact. “Purchase” is generally not a choice, and the alternative may be death or severe disability. Healthcare emergency departments are unique in having to provide service even when the consumer is unable to pay. These and other conditions often lead to misalignments between incentives and rewards (62, 63). Thus, it is no coincidence that the most significant progress in transformation has been made by a handful of integrated health systems where there is greater alignment between incentives, practice, and rewards (3, 64–66).

Despite widespread efforts over the past 20 plus years, we have seen little impact on our fragmented U.S. healthcare delivery system. Research shows that most of these efforts have been as fragmented and siloed as the delivery system itself and seldom have been translated beyond a narrow scope of practice to the delivery system more holistically (60, 67). Impediments are many.

Information sharing for care coordination is a long-standing challenge, which has become a national priority (21^st^ C. Cures Act). Failures in healthcare delivery practices for care coordination alone accounts for approximately 10–20 percent of variance in health outcomes (35). Individual behavior is also a driver of variance as is social determinants of health. Our healthcare system simply was not designed for preventing and managing chronic conditions that account for 75–80 percent of U.S. healthcare costs. It was not designed to integrate care of chronic behavioral and medical health conditions (35).

Addressing healthcare delivery problems requires convening many stakeholders with often competing interests. Bringing together siloed organizations and competitive entities with a focus on the bigger, system-wide picture for consumer-centered coordinated care can present a major challenge. Digital technologies offer revolutionary tools and opportunities. However, employing them effectively requires redesigning workflows, organizational relationships, and job responsibilities. Progress requires deep integration with medical practice, which has implications for system engineering, user interface design, algorithms, database design, and other technology and organizational issues. Healthcare innovation success stories reveal the extent to which factors such as culture, transition to team-based care, buy-in at all levels, roles of clinical leadership, and change in mindset are critical to achieving desired outcomes (64–66, 68, 69). Thus, due to this socio-technical complexity, one form of integrated care does not fit all; no single model will be suited to all contexts, settings, and circumstances. Careful analysis is needed. Decisions about which approaches are relevant in a particular setting must consider project goals, needs of service users, providers, other stakeholders, and available resources. Strategies need to address multiple levels for transformation.

1. Overcoming barriers to implementing what we already know about systems and delivery (evidence base) in a sustainable and timely manner. In many cases, significant gaps exist between best practice/evidence and what is delivered, and how it is delivered.
2. Generating new evidence (especially effectiveness) to close gaps in what is possible toward optimizing healthcare delivery and better coordinating patient care.

Moreover, working in the domain of transformational change involves additional challenges that go far beyond standard change management methodologies (68, 70) The organizational development research reveals critical distinctions involved in leading transformational change (68). Working at the level of transformational change, involves three critical and unique dynamics: (1) the future state cannot be fully known in advance; (2) significant change in organizational culture and in individual mindsets and behaviors are required, and (3) the change process itself cannot be tightly controlled and is difficult to manage because outcomes are uncertain, and the human dynamics are too unpredictable (68). These unique dynamics present many challenges for working within traditional scientific research methodologies and institutional management infrastructures. Addressing these challenges requires a new mindset, with a whole-systems, long-term, process perspective. Transformation is not something that can be led apart from where real “work” happens—it must be intertwined, embedded, and integrated into activities and infrastructure that drive action in healthcare organizations (68). These theoretical foundations underlie our recommendations for rethinking methodologies for transformational research.

Essentially, despite the strong advocacy for healthcare system change, no one in the U.S. has clear cut responsibility for leading it. Leadership is as fragmented as healthcare delivery itself (44). The Centers for Medicare and Medicaid's (CMS) innovation efforts exert influence power through Medicare/Medicaid policy and reimbursement regulations. Professional medical associations such as the American Hospital Association and the American Medical Association, bring specific perspectives and vested interests. Health IT vendors add yet another influential dimension. The Center for Disease Control (CDC) is focused on public health with limited capability of sharing health information within states let alone nationally. The Office of the National Coordinator for Health Information Technology (ONC) focuses primarily on convening stakeholders to address technology issues with the exception of the Meaningful Use (MU) program, which was managed in coordination with CMS. The ONC's current focus is on implementation of components of the 21^st^ Century Cures Act related to interoperability and information exchange. By design, the 2020-2025 Federal Health IT Strategic Plan (8) is broad in scope, providing strategies that span many federal departments, agencies, and offices. It focuses on meeting the electronic health information (EHI) needs of individuals, populations, caregivers, healthcare providers, public health professionals, payers, researchers, developers, and innovators. Although these and other efforts are all critical components, their role in addressing implementation at the frontlines of care is important but not sufficient. Little focus has been placed on *how* all the pieces come together at the frontlines of care delivery and how they impact the patient experience. Multiple gaps exist between evidence-based standards of care and practice. What needs to change and how do we translate it into practice, develop the evidence, and sustain it? Solutions call for the convergent team-based problem-solving approaches with high societal impact advocated by the National Academies of Engineering and Medicine and major funding agencies. However, we need to stop and consider that our traditional scientific methods may not be up to the task. Questions of appropriate scientific methodologies for convergent, team-based research remain largely unexplored. Needs include new frameworks for whole system approaches, models for holistic understanding of the complex healthcare delivery system, new paradigms for conceptualizing solutions, and a healthcare research ecosystem for transdisciplinary, evidence-based, co-creation of solutions at the frontlines of care.

**Example: 1**

“*Convergence among bio-medical, technological, clinical, and regulatory fields can create a knowledge network for precision medicine that integrates multiple sources of information. Molecular data, medical histories, information on social and physical environments, and health outcomes could be continuously updated and made accessible to the research community, health care providers, and the public. Analyzing connections between information sets (e.g., patients' genomes and their environmental exposures) would help scientists to formulate and test disease mechanisms and clinicians to develop new personalized treatments. (Source: NAS Convergence report).”*

The fragmentation and complexity of the healthcare ecosystem calls for a system level approach. Socio-technical systems (STS) science, which originated in response to challenges of understanding complex systems that are embedded in a human world (71), provides a powerful framework for analyzing issues behind the poor acceptability, uptake, and performance of many information technology-based interventions. Thus, the socio-technical framework seems particularly suitable to healthcare systems which are so dependent on complex human organizational structures. However, achieving significant and sustainable improvement will require going beyond standard discipline-centered research practices. The challenge is how to better design team-based transdisciplinary convergent research and how to close the current gaps between lab research and translation to the frontlines of care delivery.

## Research methodologies for healthcare transformation

The emerging science of healthcare delivery (HCDS) has begun addressing this complex system problem, but many challenges and unanswered questions remain (12, 14, 41, 49, 50, 52, 72, 73). In the opinion of Dr. James Weinstein, CEO and President of Dartmouth-Hitchcock Medical Center, healthcare delivery science “is the advance that is missing from healthcare reform legislation and without it will fail” (50). One way of conceptualizing HCDS is to think about it as working *on* the healthcare delivery system as opposed to working *in* the healthcare system. As its name implies, HCDS focuses on the processes and organizational structures that influence the provision of healthcare, rather than the biological sciences that have been the traditional emphasis in medical research (74).

Healthcare delivery science (HCDS) is generally defined as the study of the provision of healthcare and the development of frameworks and theories to improve health and healthcare services provided to individuals, communities, and populations (73, 74). HCDS is generally viewed as encompassing effectiveness research and implementation research, which includes evaluation of quality improvement interventions (53). Delivery science brings together concepts, methods and tools from medicine, the social sciences, public health, population health, engineering, technology, and business with the goal of improving individual and community health while reducing waste and harm. Critical to healthcare delivery science are the insights needed to understand why healthcare systems are failing to meet the needs and wants of the populations they serve, and a new set of skills necessary to manage and champion change in healthcare organizations such as hospital systems, payer organizations, clinical practices, and government agencies (24, 39, 53, 74, 75).

**Example: 2**

“*A recent article in Digital Medicine* *(**49**)* *asserts that “Artificial intelligence (AI) has generated much excitement, but relatively little impact in how healthcare is delivered.” The authors recommend that to address the problem of leveraging AI at scale, “we need to both broaden and deepen our thinking around how AI fits into the complexities of healthcare delivery. As the data and computing sciences for developing AI based solutions have matured, we now need a delivery science to bring those solutions into use in healthcare.” The issue of explainability of AI algorithms in all industries has become a major research issue. This is an integrity and user design issue that requires deep understanding of the ecosystem for which AI applications are designed.”*

Delivery science research seeks to overcome the barriers, understand the facilitators, and implement the innovations necessary to improve the process of healthcare (24). Delivery science has also been called “implementation science” or “translational research,” because the overarching goal is to translate the evidence of clinical research into the practice of clinical care (39). Innovations examined or implemented by delivery science researchers include new health IT tools that leverage integrated electronic health records, new payment models to overcome some of the misaligned incentives of the traditional fee-for-service models, and patient-centered models of care that integrate individual patient preferences and values. Conducting robust and rigorous research to improve health care delivery systems raises several novel research challenges, including developing a well-trained research workforce with a new set of research skills that are not standard features of traditional research training programs (39, 76).

How can or should researchers align these new requirements with traditional scientific hypothesis-driven research methodologies? How does transformational research differ from traditional translational research and applied research? Reports by delivery science researchers about how extremely competitive and challenging it is to secure funding for transformational projects suggest several areas of misalignment between expectations and new realities of research in the transformational domain (37, 39, 77). The United States spends more than $2 trillion on health care annually, but <0.1 % of this total (representing only 3.6 percent of the NIH budget) is currently allocated to research that is designed to improve how healthcare is delivered (39, 78).

Grant and Schmittdiel (39) identify several key similarities and differences in the competencies required for delivery science research. Shared competencies with traditional health science research include forming testable hypotheses, identifying and addressing sources of bias and confounding variables, and implementing rigorous biostatistical methods. New competencies that are not standard features of traditional research methods are summarized by Grant and Schmittdiel (39) in three broad domains:

1) Understanding how clinicians create and use healthcare data: Beyond the expertise to merge, clean, and validate large quantities of clinical data, delivery science researchers require a deep understanding of the significance of the information in the care process and how and why the data elements are created and used in the flow of healthcare delivery. Developing this understanding and potentially playing a role in changing the data collection process requires direct collaboration with the clinicians providing the patient care, the health IT programmers developing or modifying the EHR interfaces, as well as quality improvement leaders who may be establishing care goals.
2) Developing relationships with stakeholders: Successful translation of research into practice requires the effort of many stakeholders within a health care system and ultimately succeeds if clinician leaders are invested in performance improvement. Thus, effective collaboration between clinicians and researchers is essential. Aligning research, operational, and clinical agendas is critical, albeit challenging. To successfully implement change in a system as complex as healthcare, delivery scientists must be closely embedded within the workings of the system itself.
3) Resourcefulness in pursuing diverse and non-traditional funding sources: As a transdisciplinary applied field of research, healthcare delivery science does not clearly align with traditional scientific funding sources. Therefore, unless healthcare transformation is an institutional priority, delivery science projects often face challenges competing for internal funding, and competing for external funding is equally challenging.

**Figure 1:**
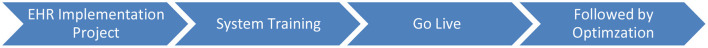
Prevalent IT change methodology for EHRs.

We can gain additional insight into the challenges of healthcare delivery science by considering the prevalent IT change methodology for implementation of electronic health record systems (EHRs) as depicted in Figure 1. It is essentially a linear top-down, siloed approach with the focus on technology requirements. One of the weaknesses of the prevailing approach is that generally 90 percent of strategy, effort, and funding go into the technology implementation phase while minimal planning and funding are allocated to optimization (i.e., meaningful use of the technology). As a result, the institution does not gain anticipated benefits and technology has minimal impact on improving the delivery of patient care, essentially layering expensive new technology on top of expensive inefficient paper processes and disgruntled clinicians burdened by the additional work with few benefits (79).

**Figure 2:**
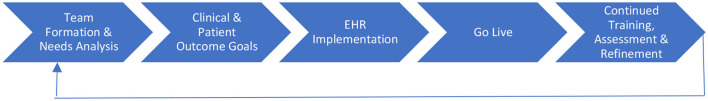
Healthcare delivery science methodologies.

In contrast, research shows that institutions that realize the greatest benefit from new technology take a far more integrated approach from a holistic, system perspective (13, 53, 80–82). Rather than starting with the technology, they focus on the clinical goals they hope to achieve, which include clear, specific improvements in patient care (the end in mind). Workflows and care pathways are redesigned in parallel with the technology installation. Training goes beyond just EHR functionality to encompass changes in workflow and procedures and often job redesign. As shown in Figure 2, the process involves a much more bottoms up approach that engages clinicians and staff at multiple levels including quality improvement —essentially a transdisciplinary, convergent design science approach. It also generally results in an EHR system configured far more effectively in alignment with practice goals.

In summary, barriers to healthcare delivery science research identified in the literature include the following: (53, 83).

- Limited funding (77).
- Insufficient leadership support (44).
- Lack of engagement between operational and research leaders.
- Limited pools of research expertise / limited access to research expertise by clinicians.
- Lack of pathways to identify and develop ideas.
- Confusion about how to prioritize opportunities.

To address these barriers, a convergence approach is critical to advance both the science and technology research as well as preparing the future workforce (53). Researchers Lieu and Madvig (53) also underscore the critical role of industry in taking advantage of the convergence opportunities moving forward. They argue that a convergence approach is critical to both advanced science and technology research as well as preparing the future workforce. Two highly relevant research methodologies associated with healthcare delivery science are design science (also called design thinking) and value co-creation (69, 84, 85).

Design science has its roots in engineering and can be defined as “an applied research and innovation framework that prioritizes empathy for users of a service or product, involves highly diverse and collaborative project teams, and encourages an action-oriented rapid prototyping of user-derived insights rather than top-down hypotheses” [(86) p. 12]. Design science and behavioral science are foundational in the information systems discipline, positioned as it is at the confluence of people, organizations, and technology (84). Design science is especially appropriate for addressing complex problems in which existing practice paradigms do not work well, requiring whole new approaches to a problem, which makes it a good fit for research in the domain of transformational change (87, 88).

Design science methodologies differ from traditional science in several distinct ways (86). It is transdisciplinary convergent problem driven in contrast to traditional science top-down hypothesis driven methodologies. Scientists put more emphasis on analysis of pre-formed hypotheses or discipline-based, theory-driven solution approaches whereas design scientists emphasize synthesizing information and ideas from many different sources, in search of new and unconventional solutions. Design science offers a framework for orienting diverse project teams around problems, as they exist within, and are experienced by individuals and communities, rather than around individual expertise, past practice, prevailing paradigms, or organizational structures (86).

Design science also differs from traditional process improvement methodologies in distinct ways: design science is most applicable early in the innovation process when problems are not well-defined, or it has become clear that current attempts to solve a problem are not working. Whereas, process improvement is most valuable when problems and possible solutions are less abstract and more relevant to current day-to-day operations (86). University of California San Francisco Health's “Caring Wisely” program has demonstrated the effectiveness of using design science methodologies to engage frontline healthcare professionals in designing intervention strategies that reduce costs, enhance healthcare quality, and improve healthcare outcomes. In fact, they cite the application of design science / implementation science principles as one of the key factors in the success of the program (89).

Applying a design science research approach can lead to solutions (artifacts), which might be in the form of new implementation processes or methods, models, care pathways, algorithms, human/computer interfaces, constructs, instantiations, design theories, other innovative products, or new technical/social/informational resources (57, 87, 90–92). The artifact enables researchers to get a better understanding of the problem; re-evaluation of the problem improves the quality of the design process and so on. This build-and-evaluate loop is typically iterated multiple times before the final design artifact is completed. The methodology involves three closely related cycles of activities. The relevance cycle initiates design science research with an application context (e.g., a use cases or care pathway innovations) that provides the requirements for the research as inputs but also defines acceptance criteria for the ultimate evaluation of the research results. The rigor cycle applies past knowledge to the research project to verify its innovation. The central design cycle iterates between the core activities of building and evaluating the design artifacts and processes of the research. Ultimately, the research team will be aiming for innovative solutions that improve healthcare delivery (39). To achieve this objective, innovations need to be embraced by all stakeholders (patients, clinicians, system leaders, researchers). Thus, direct collaboration among the clinicians providing the patient care, the researchers and programmers developing or modifying technology solutions and interfaces, and the quality improvement leaders establishing care goals is essential. In accordance with the learning healthcare system concept (24, 57, 58), implementation of solutions will continue to be evaluated, refined, and extended with the longer-term aim of transforming healthcare delivery to a more consumer centric, better coordinated interorganizational healthcare ecosystem that will extend the “health-span” of the population. The concept of health-span, defined as the length of time patients are able to maintain their health without the need for hospitalization or other intensive care treatments, relates to the paradigm shift to managing populations of patients with chronic illnesses.

Another relevant methodology is the focus on value co-creation. The concepts of value chain (93) and value co-creation (94) have been studied extensively in the business and service industry literature. Value co-creation is a central concept of Service-Dominant (S-D) logic (95), which views service industries such as healthcare as complex service ecosystems. The ecosystem perspective recognizes the holistic dynamics of complex systems, and how resources are integrated at the various system levels (micro-meso-macro). Value co-creation is generally defined as “benefit realized from integration of resources through activities and interactions with collaborators in the customer network” (96). In S-D logic, value is co-created by actors when resources are used and combined in different ways. In this sense, all actors in the customer network are resource integrators, active participants in value co-creation, connected together in embedded systems of service exchange (97). Thus, collaboration is essential (98), as actors interact to increase resource density, improve the set of resources available to them and increase the value created (99).

The next section discusses implications for rethinking methodologies for research in the domain of transformational change (i.e., societal impact) and implications for funding priorities.

## Changing the research paradigm: new models for the science of healthcare delivery and research funding

Research institutions and funding agencies have recognized the need and have made a notable shift in priorities to incorporate transdisciplinary team-based convergent research with societal impact into their funding programs. This shift is reflected in multiple recent program solicitations issued by NSF, NIH, and others. The rationale and benefits are well documented in the National Academies literature, but have we moved far enough in examining how best to adopt or adapt research methodologies to fully realize the strengths of deep convergent thinking? There seems to be an implied assumption—if not expectation—that traditional science research methods still apply. The literature on HCDS, design science, and value co-creation as well as feedback on grant proposals also suggest that reviewers expect to see traditional science methods and may be leery of more recent methodologies such as design science (4, 37, 53, 100).

However, a growing body of literature is suggesting the need for new research approaches (4, 8, 18, 37, 53, 69, 86, 89, 101). In translational science, evidence suggests that the traditional linear process of developing interventions–from proof-of-concept and efficacy to effectiveness and implementation trials–may be contributing to the lack of progress in healthcare delivery transformation as well as inequities in care delivery (4, 18). This conventional linear process embodies an inherent dichotomy between the design context and the implementation context, which leads to interventions that clinicians at the point-of-care do not find feasible, acceptable, and useful [(4). p. 3].

So, the question becomes how can we develop a scientifically rigorous research process to bridge this inherent dichotomy between lab science and translation to practice—a process that on average requires seven or more years and often turns out not to be sustainable or scalable? And more broadly, how can we develop scientifically rigorous research methods to address the realities of designing, testing, and validating transformational change in healthcare delivery as well as support the concept of “learning healthcare organizations” called for in the seminal Institute of Medicine reports (40)? In their research on reframing implementation science to address inequities in healthcare delivery, Bauman and Cabassa (4) discuss the need to restructure grants and contracts to build time, resources, and infrastructure in the initial stages of a project to develop true partnerships between researchers and vulnerable communities.

In summary, the discussion in the prior section suggests that extending healthcare delivery science methodologies to bridge this inherent gap between research and practice requires several significant paradigm shifts from traditional science methodologies as summarized in Table 1 These paradigm shifts and the HCDS literature, in turn, suggest several critical success factors for closing the gaps between research and translation at the frontlines. A growing body of evidence indicates that research needs to move out of the lab and closer to the frontlines (4, 13, 36, 49, 53, 68, 73, 89, 100, 102–104). These critical success factors include:

- An ecosystem, holistic (systemic) perspective.
- Transdisciplinary convergent team-based, design science approach.
- Co-leadership of projects by researcher and clinician.
- Begin with convergent transdisciplinary research to gain new insights into the problem (medicine, engineering, technology, behavioral health, social sciences, population health).
- Hypotheses generated based on transdisciplinary convergent problem solving instead of an extension of prior research.
- Co-creation of solutions at the front lines (103, 104).
- Prior research helps inform solution evaluation.
- Optimize around patient experience rather than institutional operations.
- Ensuring equity in the delivery of care.
- Integration of the learning healthcare system concept (105).
- Sufficiently long-term view.

**Table 1:** Research paradigm shifts inherent in healthcare delivery science.

**FROM**	**TO**
Research *about* the frontlines of care	• Research *at* the frontlines of care.
• Transdisciplinary convergence in thinking	• Transdisciplinary action for doing.
• Translating proof-of-concepts	• Co-creation of solutions
• Short term perspective (2–3 years)	• Long term perspective (10+ years)
• Focus on initial implementation	• Focus on sustainability and scalability
• Evidence-based medicine	• Learning healthcare system
• Hypothesis generation based on prior research	• Hypothesis generation based on transdisciplinary convergent problem solving
• Team roles based on discipline	• Building team convergence as an intentional project activity
• Incremental innovation	• Transformational change

The proposed transformational research methodology for healthcare delivery science (**Figure 4**) is based on a transdisciplinary synthesis spanning design science, implementation science, translational research, transformation change theory, systems theory, service-dominant logic, and team-based transdisciplinary convergent research. As a transdisciplinary, socio-technical research methodology, design science makes a critical shift in the research focus from describing and explaining the existing world to helping shape it (90, 106).Thus it is particularly useful for complex system research problems such as those involved in designing for digital transformation. It provides a structured framework for iterating solutions, implementing, evaluating, and guiding additional research.

The methodology takes an integrative approach to incorporate transdisciplinary team building and analysis at the front end. Starting with convergent problem analysis is intended to build transdisciplinary insight into the complex dynamics of the problem ecosystem, which in turn, drives thinking about desired outcomes for change, and the gaps in patient needs, expertise, and research between current practice and desired outcomes. It strives to close the inherent disconnect between research and practice by moving the research out of the lab closer to the frontlines to co-create solutions. Evaluation goes beyond assessing whether a solution achieved the desired improvement in clinical process or outcome to assessing impact on the system as-a-whole. It includes criteria such as how well or poorly the solution was implemented, practice adoption rates, scalability, sustainability, and impact on patient experience and outcomes (106). An ecosystem perspective is crucial for researching the holistic dynamics of complex systems, which requires moving away from an institution-centric perspective to focusing on the holistic context of a complex world (107). This ecosystem perspective makes complex contexts such as healthcare more understandable by applying systems-level thinking (37, 44, 103, 108). Ecosystems are dynamic, constantly changing on multiple system levels (micro, meso, and macro). While these levels are different, they are interdependent within the whole system. Thus, changes in one unit or level have ripple effects (sometimes unanticipated) across other levels. The implication is that making changes in one unit or level requires corresponding change on other levels to achieve the anticipated benefits. This is a major gap in the prevalent model of siloed disciplinary innovation, which often results in failure to attain sustainability.

**Figure 3:**
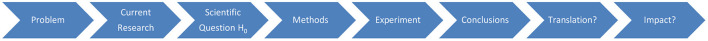
Traditional science research methodology.

**Figure 4:**
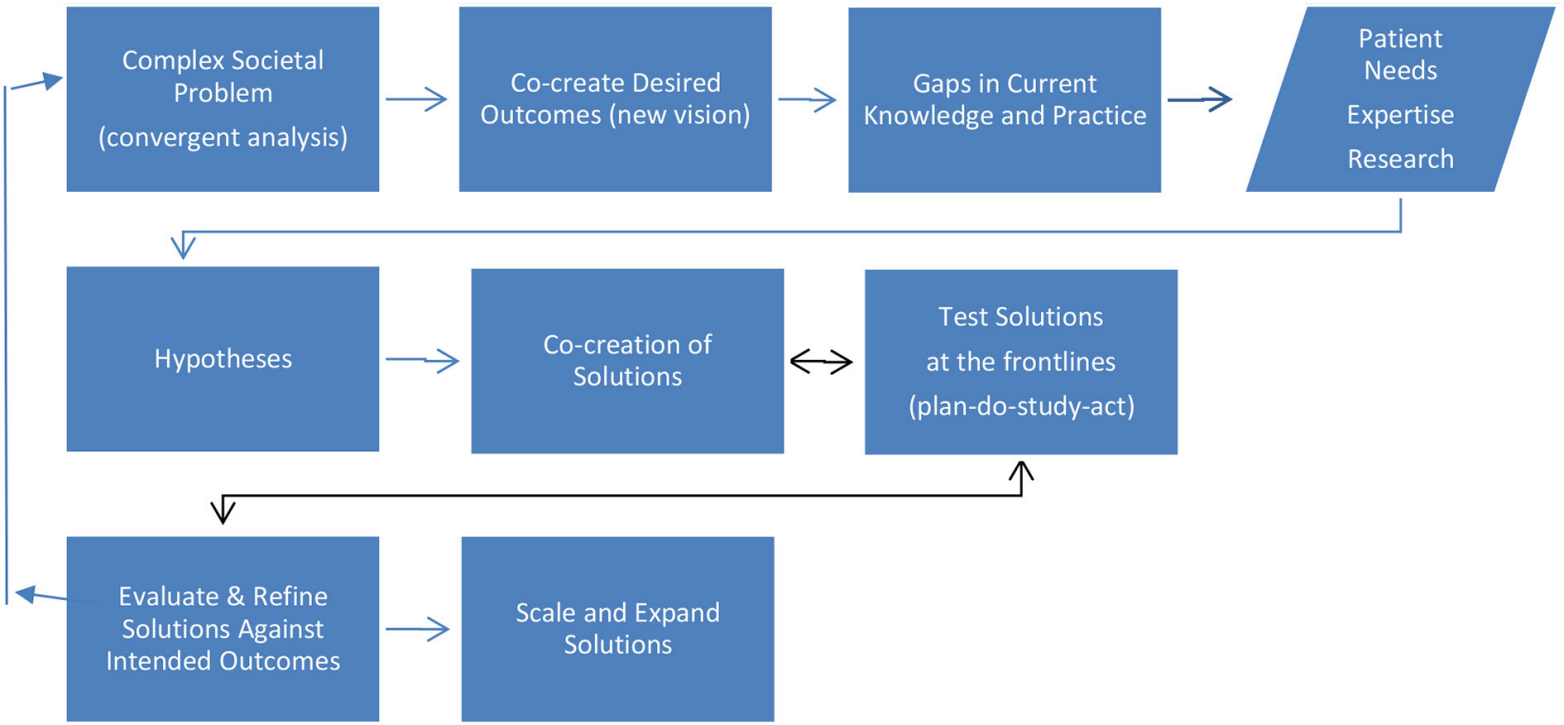
Transdisciplinary, convergent transformation research.

Figures 3, 4 contrast the traditional science research methodology with the proposed convergent team-based transformational research methodology. Evidence indicates that the traditional linear process for developing interventions, from proof-of-concept and efficacy to effectiveness and implementation trials, often leads to implementation gaps due to the inherent “disconnect between the design context and the implementation context” (4, 18, 109). This gap often leads to treatments or delivery processes that are not feasible, acceptable, and useful with the realities of routine practice (4). This disconnect may be most pronounced in underserved communities further contributing to inequities in care delivery.

In contrast to the linear methodology of the traditional science research model, the proposed methodology is more dynamic, integrative, and holistic (systemic) in approach. It is intended to shift more research to the core processes that drive healthcare delivery. It takes into account the unique dynamics of transformational change with its associated changes in organizational culture and individual mindset and behavior. It addresses people, process, technology, and structure. Hypothesis development is based on convergent analysis of complex problems rather than the more traditional theory-driven hypotheses or theory-driven solution approaches.

The proposed research methodology for convergent transdisciplinary transformation research provides a more holistic ecosystem perspective for healthcare delivery science research. It advances the science beyond current siloed innovation approaches to address system level transformation. The problem driven design science approach starts with convergent analysis of the status quo to develop deeper insight into the symptoms and root causes of the problem. Transdisciplinary research teams span multiple disciplines, such as medicine, information technology, engineering, behavioral sciences, and transformational change. Co-creation at the frontlines involves clinicians, patients, and researchers with the goal of closing the gap between lab research and translation to practice. The methodology is intended to provide additional rigor and structure to the research process to develop reliable evidenced-based interventions that are responsive to the implementation context and meet the needs of both patients and clinicians. It is hoped that the methodology will contribute to making healthcare delivery science research grant proposals more competitive with traditional disciplinary research proposals and help reviewers and decision makers evaluate funding requests.

**Figure 5:**
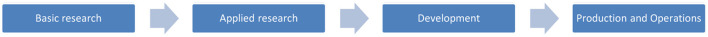
Prevailing R&D model of technology transfer.

Achieving this goal for funding parity with traditional basic science research still faces significant challenges (4, 6, 7, 18, 24, 45, 52, 106, 110). Since the mid-20^th^ century, funding decisions by NSF and other federal agencies have been guided by longstanding distinctions between basic research (understanding) and applied science (use), which characterized them as empirically separate (111). This distinction reflects the belief that basic and applied research are separate endeavors, pursued by different people “with different gifts and different interests,” which over time evolved to giving priority to the discoveries of basic science. Ultimately, these conceptualizations of research have evolved to today's prevailing research and development (R&D) Model of technology transfer, as illustrated in Figure 5 (111).

The underlying assumption is that basic science advances are the principal source of technological innovation, and this has become the prevailing paradigm of the relationship of science to technology (111). However, this linear model, which is viewed sequentially extending from basic research to new technology (technology transfer to industry), is increasingly being brought into question. Researchers such as Douglas Stokes, author of *Pasteur's Quadrant: Basic Science and Technological Innovation*, challenge the basic premise of scientific discovery being the primary driver of technological innovations. Stokes maintains that the annals of science suggest that this premise has always been false to the history of science and technology and cites numerous examples of a notable reverse flow, from technology to science. The argument being made is that basic and applied research are not necessarily opposite ends of the research continuum, but complementary and increasingly in today's technological world, integrated and dynamic. Nonetheless, the longstanding paradigm of basic vs. applied research still tends to influence evaluation and fundability of research proposals and publication of research results. So, the question becomes, what changes are needed in funding policies and practices to increase the effectiveness of transdisciplinary, team-based convergent research and support the proposed research methodology?

Discussion and recommendations about grant funding for so-called applied research methodologies (use related such as design science, transformational change, business process reengineering) are sparse in the research literature. Issues and challenges discussed in preceding sections suggest several requirements that should be taken into consideration in designing future research funding programs to promote convergent research and transformation of the U.S. healthcare delivery system. Research methodologies and funding should:

- Align with the longer timeframes required to achieve transformational change (8–10 years).
- Support transdisciplinary team-based convergent research processes, including for team leadership (Research/Clinician Co-PIs).
- Recognize the socio-technical nature of IT-based innovation, design science, and translational (applied) research.
- Incorporate problem analysis and solution development as part of the convergent research process and methodologies such as design science, iterative hypotheses development, and the learning healthcare system concept.
- Recognize the unique characteristics of transformational change: where the future state cannot be fully known in advance, requires significant cultural and mindset change, and cannot be tightly controlled because outcomes are uncertain and human dynamics are complex.
- Focus on outcomes and impact as part of the contribution to new knowledge.
- Support partnerships at the frontlines through strategies such as Researcher/Clinician Co-PI roles, co-creation of solutions not just translation, creative academic/industry relationships.
- Consider issues and goals from multiple perspectives including: academic, funding agencies, industry, patient experience.
- Support systemic approaches (breaking down the silos and fragmentation of care) that consider impact at the micro, meso, and macro levels and long-term sustainability. Identify all stakeholders in care delivery processes (not just clinician and patients). Define care pathways across the entire continuum of care from the patient perspective (not just the unit or institution perspective).

The proposed research methodology holds promise for increasing the opportunities and impact for research collaborations between university researchers and the healthcare industry. Our educational institutions comprise a reservoir of resources and researchers in the health sciences, medicine, pharmacy, public health, social sciences, engineering, and computing and technology on which to draw. However, like our care delivery system, they are fragmented and operate predominantly in disciplinary and institutional silos, which limit translation to systemic problems like healthcare delivery. The complex system problem of healthcare delivery transformation requires a sustained transdisciplinary, convergent design science approach; there is no “quick fix.” Addressing it requires rethinking how our national research and funding infrastructure can support formation of a more effective convergent team-based transdisciplinary research ecosystem to enable it. Making this problem a national research priority could make a significant impact, especially now as our nation has begun implementing data standardization and national health information exchange capabilities called for in the 21^st^ Century Cures Act. Achieving the aims for a fully connected healthcare system that empowers patients, caregivers, and their healthcare providers to access, exchange, and use digital health information to better coordinate care could be a game changer. We invest heavily in research for working *in* the system; much greater investment is now needed for working *on* the system.

## Data availability statement

The original contributions presented in the study are included in the article, further inquiries can be directed to the corresponding author.

## Author contributions

The author confirms being the sole contributor of this work and has approved it for publication.

## Conflict of interest

The author declares that the research was conducted in the absence of any commercial or financial relationships that could be construed as a potential conflict of interest.

## Publisher's note

All claims expressed in this article are solely those of the authors and do not necessarily represent those of their affiliated organizations, or those of the publisher, the editors and the reviewers. Any product that may be evaluated in this article, or claim that may be made by its manufacturer, is not guaranteed or endorsed by the publisher.
